# The Action of Hydrocyanic Acid on Iodine

**Published:** 1848-01

**Authors:** 


*The Action of Hydrocyanic Acid on Iodine.*—Dr. HOPE, of Booth, near Liverpool, after remarking that if we take a solution of either the simple or compound tincture of iodine, of the Pharmacopœia, we can easily detect its presence with starch, and that if to this solution we add sulphuric, nitric, hydrochloric, tartaric, or acetic acid, we shall find they have no influence on the colour, nor will they interfere with the action of starch as a test, adds, if, however, we take a solution of hydrocyanic acid, (Scheele's strength,) fifteen minims to one ounce of water, we shall find a very different result; for if from fifteen to twenty minims of the tincture of iodine, simple or compound, be added guttatim, it will be immediately bleached, the water remain perfectly colourless, and no trace of the iodine be detected. Dr. Hope concludes his communication by putting the following queries:

"1. Is the action reciprocal, and how is the change effected?

"2. How far would it be useful to administer iodine in cases of poisoning by prussic acid?

"3. In case of an overdose of iodine, or a liniment containing it, being swallowed, would any good result be obtained by giving the hydrocyanic acid?"—*London Lancet.*

